# Chemical Count: Quantifying Exposures in Pregnant Women

**DOI:** 10.1289/ehp.119-a258b

**Published:** 2011-06

**Authors:** Kellyn S. Betts

**Affiliations:** **Kellyn S. Betts** has written about environmental contaminants, hazards, and technology for solving environmental problems for publications including *EHP* and *Environmental Science & Technology* for more than a dozen years

A nationally representative assessment of pregnant women’s exposure to 163 chemicals reveals what the authors term “ubiquitous exposure to multiple chemicals during a sensitive period of development” **[*EHP*** **119(6):878–885; Woodruff et al.]**. The new study is based on samples collected and analyzed as part of the National Health and Nutrition Examination Survey (NHANES) 2003–2004.

The researchers assessed data for 268 pregnant women between the ages of 15 and 44. Chemical analytes assessed included metals, perfluorinated compounds, organochlorine pesticides, organophosphate insecticide metabolites, phthalates, polybrominated diphenyl ethers (PBDEs), polycyclic aromatic hydrocarbons (PAHs), phenols, polychlorinated biphenyls (PCBs), dioxin-like chemicals, perchlorate, triclosan, and volatile organic compounds. Not all analytes were measured in all women.

The study showed the pregnant women had widespread exposure to substances banned decades ago as well as contemporary contaminants. Several of the chemical analytes assessed were detected in 99–100% of the pregnant women. There was substantial variation in the levels of individual analytes to which pregnant women were exposed. Most notably, the difference between the geometric mean and 95th percentile for phthalates and one PBDE, BDE-153, varied by more than an order of magnitude. More research is needed to identify the major sources of exposure to these compounds among pregnant women and the general population, the authors say.

Although no health effects were assessed as part of this study, levels of many chemicals detected—including mercury, phthalates, PBDEs, and PCBs—were similar to those associated with adverse reproductive and developmental effects in epidemiologic studies. The study also showed that many women were exposed to multiple chemicals that may contribute to the same adverse outcomes. For example, perchlorate, PCBs, PBDEs, and triclosan have all been associated with changes in maternal thyroid hormones, whereas mercury, lead, and PCBs can all harm the developing brain.

The authors point out that exposure to multiple chemicals that act on the same adverse outcome can have a greater effect than exposure to an individual chemical. The National Academy of Sciences recommends accounting for multiple exposures, as well as exposures that occur during sensitive periods of development, in order to improve assessment of chemical risks across the U.S. population.

